# The Binding of Learning to Action in Motor Adaptation

**DOI:** 10.1371/journal.pcbi.1002052

**Published:** 2011-06-23

**Authors:** Luis Nicolas Gonzalez Castro, Craig Bryant Monsen, Maurice A. Smith

**Affiliations:** 1 Harvard School of Engineering and Applied Sciences, Cambridge, Massachusetts, United States of America; 2 Harvard-MIT Division of Health Sciences and Technology, Cambridge, Massachusetts, United States of America; 3 Center for Brain Science, Harvard University, Cambridge, Massachusetts, United States of America; University College London, United Kingdom

## Abstract

In motor tasks, errors between planned and actual movements generally result in adaptive changes which reduce the occurrence of similar errors in the future. It has commonly been assumed that the motor adaptation arising from an error occurring on a particular movement is specifically associated with the motion that was planned. Here we show that this is not the case. Instead, we demonstrate the binding of the adaptation arising from an error on a particular trial to the motion experienced on that same trial. The formation of this association means that future movements planned to resemble the motion experienced on a given trial benefit maximally from the adaptation arising from it. This reflects the idea that actual rather than planned motions are assigned ‘credit’ for motor errors because, in a computational sense, the maximal adaptive response would be associated with the condition credited with the error. We studied this process by examining the patterns of generalization associated with motor adaptation to novel dynamic environments during reaching arm movements in humans. We found that these patterns consistently matched those predicted by adaptation associated with the actual rather than the planned motion, with maximal generalization observed where actual motions were clustered. We followed up these findings by showing that a novel training procedure designed to leverage this newfound understanding of the binding of learning to action, can improve adaptation rates by greater than 50%. Our results provide a mechanistic framework for understanding the effects of partial assistance and error augmentation during neurologic rehabilitation, and they suggest ways to optimize their use.

## Author Summary

Einstein once said: “Insanity is doing the same thing over and over again and expecting different results”. However, task repetition is generally the default procedure for training a motor skill. This can work because motor learning ensures that repetition of the same motor task will lead to actions that are different, as errors are reduced and motor skill improves. However, here we show that task repetition, although not “insane”, is inefficient. The machine learning algorithms used to control motion in robotics adapt the movement that was actually made rather than the planned movement in order to assure stable learning. In contrast, it had been widely assumed that neural motor systems adapt based on the planned rather than the actual movement. If this were the case, task repetition would be an efficient training procedure. Here we studied the mechanisms for motor adaptation in humans and found that, like in robotic learning, the adaptation that we experience is associated with the actual movement. This finding led to the design of an improved training procedure that avoids task repetition. Instead, this procedure continually adjusts the movement goal in order to drive participants to experience the correct movement, even if initially by accident, leading to an over 50% improvement in the motor adaptation rate.

## Introduction

When learning to swim, the proper stroke motion is usually taught on the pool deck. Although a student might seem to have mastered this motion on dry land, upon entering the water she will have difficulty in accurately reproducing it underwater. However, after many laps, the student eventually learns to produce the pattern of motor output that leads to the proper stroke motion while swimming. This learning occurs via the formation of internal models of the physical dynamics experienced which allow the programming of movement to contend with the dynamics of the environment [1]–[4]. These internal models have been shown to predict the dynamics of the environment as a function of motion rather than as a function of time [5]–[8] – a strategy that makes sense in light of the viscoelastic and inertial physics of our own limbs and the objects we interact with. Consequently, the neural plasticity which underlies this learning must establish associations between motion state (i.e., position and velocity vectors) and motor output which can counteract environmental forces. Although the existence of these associations has been well established, the mechanism by which they form is not yet understood.

How does this state-dependent learning arise during the course of motor adaptation? One possibility is that on individual trials, an internal model of the environment is updated based on a combination of the errors experienced and the motion plans that led to those errors. Another possibility is that internal models are updated based on errors experienced in combination with the actual motion states associated with those errors. It is remarkable that previous work on motor learning in neural systems has widely assumed the former [4], [9]–[16], despite the fact that direct evidence for this hypothesis is scant. The idea that learning is associated with the motion that was planned (plan-referenced learning) is especially pervasive in the learning rules of the algorithms that have been proposed to model the process of adaptation in the neuromotor learning literature [4], [9], [11]–[12], [15], however it is difficult to find work that addresses the validity of this assumption, explores its implications or provides a clear rationale for its use.

The machine learning community has developed, in parallel, a series of algorithms for updating internal models in robotic systems. Interestingly, these algorithms almost uniformly involve learning rules in which internal models are updated based on a combination of the errors experienced and the actual motion associated with those errors (motion-referenced learning) rather than the motions that were planned [17]–[21]. The choice of these learning rules is grounded in the idea that adaptive changes should be provably stable in the sense that, under a set of reasonable assumptions, updated internal models should never result in worse performance [17]–[21]. Here we ask the question: Do the associations between motor output and motion state formed during human motor learning arise from adaptation based on planned or actual motions? The answer to this question is important not only for theories of motor control, and issues of stability during learning, but also because knowledge of how associations are formed during motor learning can be leveraged to improve the efficiency of training procedures.

Motor adaptation can be described as the process of tuning motor output to reduce the errors between plan and action. Thus the associations between motion state and motor output formed during this process result from the way that responsibility for these errors is assigned. This is known as a credit assignment problem. This problem can be posited as the task of assigning blame after an error is experienced to the set of actions that would be most likely to give rise to similar errors in the future. This set of actions could then be modified in order to improve performance in subsequent trials. Viewed in this way, the distinction between plan-referenced learning (PRL) and motion-referenced learning (MRL) corresponds to whether the blame for motor errors should be assigned to the planned versus actual motion. Consequently, the amount of adaptation on a given trial will be determined by the magnitude of the error, however the location of the adaptation (which future motions will benefit from the adaptation) will be determined by the credit assignment mechanism. Here we studied the generalization of motor adaptation to untrained conditions in order to elucidate the credit assignment mechanism used by the CNS, and then used our understanding of this mechanism to design a training paradigm that takes advantage of it to improve the efficiency of motor adaptation.

## Results

### What are the implications of different credit assignment mechanisms in the CNS?

**Figure 1:**
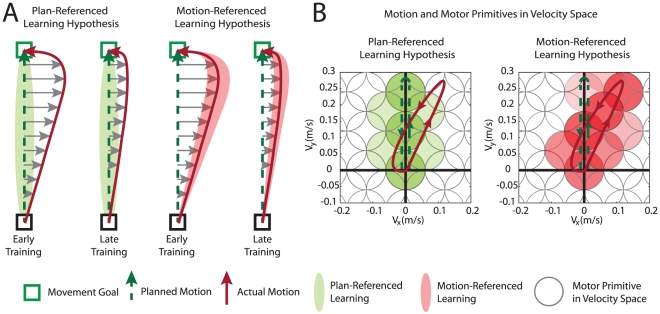
Two hypotheses for credit assignment during motor adaptation. (**a**) Illustration of planned (green dashed line) and actual (solid red line) trajectories for early (left) and late (right) movements during adaptation to a velocity-dependent curl FF (grey arrows). Plan-referenced learning (PRL) would lead to adaptation associated with the planned motion (green dashed line). In contrast, motion-referenced learning (MRL) would lead to adaptation associated with the actual motion (solid red line). The green- and red-shaded regions represent the space of motions that would experience the greatest amount of adaptation under PRL and MRL, respectively. (**b**) Illustration of the adaptation of velocity-dependent motor primitives under PRL and MRL for early training. Here each 2-dimensional, Gaussian-shaped motor primitive is represented by a gray circular contour at its half-*σ* point (σ = 0.12 m/s from [12]). The preferred velocities (centers) of these motor primitives are tiled across velocity space as shown. Note that the planned and actual arm motions (green dashed line and red solid line) are replotted in velocity space here. The interior of the circle representing each motor primitive is colored with an intensity proportional to the activation induced by the adaptation resulting from the illustrated trial. Under PRL (left panel) this activation is greatest for motor primitives which neighbor the motion plan in velocity space (green shading), whereas under MRL this activation is greatest for motor primitives that neighbor the actual motion (red shading).

The adaptations that would occur at different stages of training for reaching arm movements in a velocity-dependent force-field (FF) for the PRL and MRL credit assignment hypotheses are shown in Figure 1. The green shaded region around the planned motion – which is essentially straight toward the target for short (10 cm) movements [22] – represents the space of future motions which would benefit from the adaptation to the greatest degree under PRL (Figure 1A). Alternatively, each red shaded region represents the space of future motions which would benefit maximally under MRL. A more direct visualization of the adaptive changes predicted by each credit assignment hypothesis can be made by representing motion and the resulting adaptation in velocity-space rather than position-space, since the adaptation to the velocity-dependent dynamics studied in the current series of experiments is believed to be mediated by an internal model largely composed of velocity-dependent motor primitives [8], [10], [12]–[13], [23]. These primitives are the learning elements which contribute to the compensatory motor output (i.e., compensatory force) in a velocity-dependent manner. Figure 1B shows how individual motor primitives would adapt based on PRL versus MRL credit assignment early on in training. Here each circle represents a single motor primitive (centered at its preferred velocity) with a color intensity denoting the amount of adaptation that would arise from the illustrated trial. The left and right panels of Figure 1B show the adaptations predicted by PRL (green) and MRL (red), respectively. As in Figure 1A, adaptation is centered on the planned motion for PRL and centered on the actual motion for MRL.

As training proceeds over the course of several trials, the activation levels of the adapted primitives would continue to increase. This continued increase in activation (not illustrated) leads to increased compensatory force, resulting in greater compensation of the external dynamics and thus straighter trajectories. Note that the adapted primitives would be noticeably different for the two credit assignment hypotheses early in training, but would overlap late in training as force compensation increases and the planned and actual motions converge as illustrated in Figure 1A.

### Generalization after exposure to interfering force-fields reveals motion-referenced learning

Given the different implications that the PRL and MRL credit assignment mechanisms have for motor adaptation, we can assess which one is favored by the CNS by asking a simple question: After training, which motions gain the most benefit from the induced adaptation? The motions that were planned or the motions that were experienced? Since the mechanism for credit assignment determines which motions will benefit from adaptation on a particular trial, we studied how motor adaptation to a single target direction generalizes to neighboring motion directions. If a particular motion is trained, the pattern of generalization can be viewed as a record of the history of credit-assignment for the errors experienced during a training period. Specifically, the amount of generalization in the directions neighboring the trained movement constitutes the set of actions that the motor system believes should be adapted based on the history of errors experienced. Therefore, PRL and MRL should give rise to different patterns of generalization.

**Figure 2:**
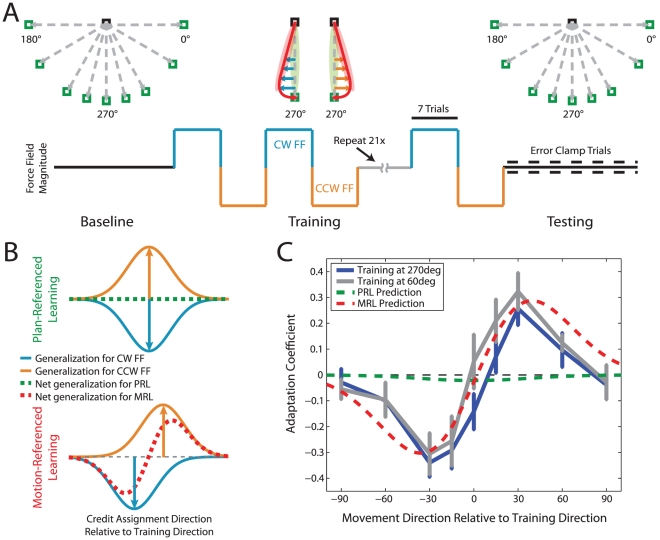
Generalization after exposure to interfering force-fields reveals motion-referenced adaptation. (**a**) Experiment schematic. After a baseline period where subjects performed movements in nine different directions, subjects received training for a single target location (the central one) with alternating blocks of 7±2 force-field trials in CW (blue) and CCW (orange) FFs as illustrated. After training, generalization of the force-field compensation was tested along the nine original directions practiced during the baseline period (see Materials and Methods for details). (**b**) Credit assignment predictions. If the motor primitives that are adapted during training are centered at the desired movement direction – as specified by PRL – the exposure to opposite force-fields would lead to opposite generalization patterns for the CW and CCW FFs (orange vs. blue in the top panel) that would essentially cancel one another leading to a near-zero net generalization pattern (green dashed line). In contrast, if the motor primitives that are adapted during training are centered at the actual movement directions – as specified by MRL – the exposure to the CW and CCW force-fields would lead to individual generalization patterns for these FFs that are misaligned (orange vs. blue in the bottom panel). The sum of these misaligned generalization patterns would result in a bimodal generalization pattern (red dashed line). (**c**) Experimental results. In two different experiments (one where subjects were trained at 270° – blue line – and another where subjects where trained at 60° – grey line) the patterns of generalization obtained appear consistent with motion-referenced learning (red dashed line, r = 0.92 (270° data) and r = 0.93 (60° data)) but inconsistent with plan-referenced learning (green dashed line). The error bars represent standard errors.

In order to cleanly distinguish between these hypotheses, we designed an experiment in which the planned motion and the actual motion were maintained to be distinct from one another during the entire dataset so that the patterns of generalization predicted by PRL vs. MRL would be very different from one another. This is a challenge because, training a motor adaptation generally results in improved performance such that the actual motion converges onto the planned motion, and such a scenario could hamper the ability to clearly distinguish between the PRL and MRL hypotheses. Thus, we designed an experiment in which actual motion would not converge onto planned motion during the course of training, resulting in enduring differences between the predictions of these two hypotheses. To accomplish this, subjects were exposed to a training period consisting of short, successive blocks of movements towards a single target location with a force-field (FF) that alternated between clockwise (CW) and counterclockwise (CCW) directions from block to block (see Figure 2A). The magnitudes of the CW and CCW FFs were, respectively, 9 and −9 N/(m/s). In these FFs, the peak force perturbations were 2.7 and −2.7 N, respectively, for an average movement with a peak speed of 0.3 m/s. The FF blocks were short enough (7±2 trials) that neither the CW nor the CCW FF could be learned very well before unlearning with the opposite FF occurred. After subjects were exposed to a number of these interfering FF cycles, we measured the generalization of adaptation to untrained movement directions with error-clamp (EC) trials (see Materials and Methods for details).

The predictions of PRL and MRL are strikingly different for this experiment. For the PRL hypothesis, since the adaptation is associated with motor primitives centered at the same target direction for both FFs (Figure 2B top panel, blue and orange traces), the balanced exposure to these opposite FFs would lead to cancellation of the CW and CCW FF learning resulting in near zero adaptation at the trained target direction and the adjacent directions (Figure 2B, dashed green trace). Note that although target locations are identical between CW and CCW FF trials, the actual movement directions differ. The CW FF perturbs motion towards smaller movement angles whereas the CCW FF does the opposite. Therefore, MRL predicts that smaller movement angles would be preferentially associated with adaptation appropriate for the CW FF (blue trace in the bottom panel of Figure 2B), whereas higher movement angles would be preferentially associated with adaptation appropriate for the CCW FF (orange trace in the bottom panel of Figure 2B). This would lead to the bimodal pattern of generalization illustrated in Figure 2B (red dashed trace).

We trained one group of subjects in this FF interference paradigm at a target location of 270°. We found that target directions smaller than the training direction consistently display generalization appropriate for the CW FF (negative) whereas target directions greater than the training direction display generalization appropriate for the CCW FF (positive). This is consistent with the bimodal generalization pattern predicted by MRL (compare the blue and red traces in Figure 2C: r = 0.92, *F*(1,7)  = 36.87, *p*<0.001) and quite different from the essentially flat pattern predicted by PRL (green trace). Correspondingly, we found the adaptation levels at the target directions corresponding to the peaks of the predicted generalization pattern (−30° and +30°, see Figure 2C) to be significantly different from one another (*t*_11_ = 7.26, *p*<9×10^−6^) and from zero (*t*_11_ = 5.95, *p*<5×10^−5^ for −30°, and *t*_11_ = 3.89, *p*<0.002 for +30°). These results provide direct evidence for MRL by matching the complex pattern of generalization predicted by it.

In our experiment we balanced the direction of the FF that was presented before testing generalization, nevertheless, we noticed a small bias in the generalization function at the training direction consistent with a bias in adaptation level that we observed during the training period (see Figure S1 and Text S1). This bias is compatible with other results showing somewhat faster learning for a CW FF [8]. In order to eliminate the possibility that this bias or the target location we chose for training (270°) might have somehow contributed to the generalization pattern we observed in the data, we trained a second group of subjects in a version of this experiment that was designed to eliminate the bias and provide training at another target location (60°). We eliminated the bias by unbalancing the number of CW versus CCW FF trials in each cycle in this second group of subjects (see Text S1). We found that the close match between the pattern of generalization that these subjects displayed (Figure 2C, grey trace) and the pattern predicted by MRL persisted under these conditions (r = 0.93, *F*(1,7)  = 42.61, *p*<0.001). Correspondingly, the adaptation levels at −30° and +30° were significantly different from each other (*t*_9_ = 5.37, *p*<3×10^−4^), and significantly different from zero (*t*_9_ = 3.72, *p*<0.003 for −30°, and *t*_9_ = 4.38, *p*<9×10^−4^ for +30°). Together, these results provide compelling evidence for MRL as the mechanism for credit assignment in motor adaptation.

We note that Equations 3 and 4 used for our simulations incorporate local motor primitives that are functions of the initial movement direction (*θ*) rather than of the full time series of the velocity vectors encountered during each trial. This might seem an inappropriate choice since, as we discussed above, velocity-dependent motor primitives are thought to underlie the learning of velocity-dependent dynamics [8], [10], [12]–[13], [23]. However this approximation is a good one when movements are approximately straight, which is essentially the case for the first 400 ms of the movements considered in our study. This approximation, of course, breaks down at the end of the movement when the initial movement direction no longer describes the velocities experienced. However, the amplitudes of the velocity vectors during the end-movement correction are quite low and so the unmodeled spread of learning to the actual motion experienced in this correction phase should have relatively little effect since at low velocities, viscous dynamics have small consequences. This effect can be visualized in the left panel of Figure 1B which shows that the end-movement correction which has a velocity vector that points to the second quadrant would only excite velocity-dependent primitives near the origin under MRL.

Note that the separation of the peaks in the bimodal generalization pattern predicted by MRL (red dashed line in Figure 2C) results from the size of the errors experienced during training. Consequently, larger force-field perturbations which induce larger errors would result in greater separation between the peaks. However, the separation between the peaks (about 60°) is predicted to be greater than the separation between the average errors experienced in the two force-fields (about 25°). There are two reasons for this. The first is that more adaptation occurs on trials with larger errors than those with smaller errors, skewing the center of adaptation for each force-field outwardly from the mean experienced error. The second reason is illustrated in the lower panel of Figure 2B: When the patterns of generalization for the positive and negative force-fields are summed, resulting in a bimodal generalization pattern for MRL, the peaks of this bimodal generalization pattern (red) are separated by an even greater distance than the peaks of the positive (orange) and negative (blue) components because the amount of cancellation between these components is greater at movement directions corresponding to smaller rather than larger errors resulting in further outward skew.

Previous work has attempted to measure the generalization functions (GFs) associated with learning a single FF. MRL predicts that these GFs will be shifted toward the motion directions experienced during training. Many of these studies have estimated GFs from complex datasets using a system identification framework [10], [12]–[13]. However the implementation of this framework assumed PRL in these studies, thus preventing a straightforward interpretation of their results. In one study [24] a simpler generalization experiment was conducted, in which subjects were trained with a single FF to a single target location, after which the resulting GF was measured. Because the actual motions approached the planned motions late in training, the shifts predicted by MRL would be subtle. Furthermore, the ability to detect shifts in the generalization function was hampered by a coarse sampling of the generalization function (45°). Nevertheless, careful inspection of these GFs consistently reveals subtle shifts towards the motions experienced during training as predicted by MRL. However, it is difficult to be certain whether if the shifts observed in this study result from MRL rather than innate biases in generalization functions because only a single FF direction was studied. Innate biases might stem from biomechanical asymmetries or direction-related biases in adaptation. We therefore performed a pair of single-target, single-FF experiments in order to compare the shifts in generalization associated with opposite FFs. The results of these experiments confirm the existence of subtle but significant shifts in generalization [25]. The magnitudes and the directions of these shifts are consistent with the MRL hypothesis [25].

### Design of training paradigms inspired by the mechanism for credit assignment

**Figure 3:**
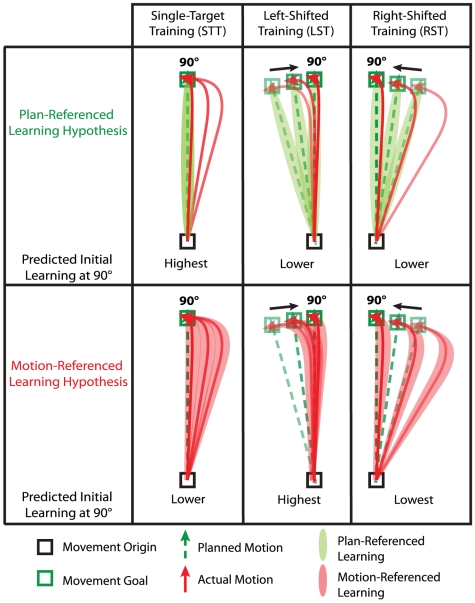
Illustration of different training paradigms under the two credit assignment hypotheses. Single-target training (STT): a single target location is presented during the training period. The PRL hypothesis predicts alignment of credit assignment across trials for STT, whereas MRL predicts misalignment. Left-shifted training (LST): targets are initially presented leftward of the desired learning direction and are brought closer to it as training progresses so that the actual motion matches the desired learning direction throughout the training period. The MRL hypothesis predicts alignment of credit assignment across trials for LST, whereas PRL predicts misalignment. Correspondingly, PRL predicts that STT will yield the greatest learning whereas MRL predicts that LST will yield the greatest learning. Right-shifted training (RST): the training targets are presented in a sequence that mirrors LST. Both the PRL and MRL hypotheses predict misalignment for RST. However, PRL predicts an identical amount of misalignment for LST and RST, whereas MRL predicts much greater misalignment for RST than LST. Note that CW FF training is illustrated in all panels.

Insights into the mechanisms for learning in the CNS can provide a platform for creating training procedures that leverage these insights to improve the rate of learning – an important goal for both motor skill training and neurologic rehabilitation. With our new understanding of how the CNS solves the credit assignment problem, we looked into the possibility of designing a novel training paradigm to take advantage of this knowledge. A key consequence of plan-referenced learning is that this mechanism for credit assignment would result in a match between what is learned and what is commanded on the next trial if the same motion plan is repeated from one trial to the next during training – like when aiming a dart at the bull's eye repeatedly. In contrast, motion-referenced learning would result in a mismatch. Motion-referenced learning, therefore, predicts that the process of training an accurate movement to a given target location in a novel dynamic environment would be inefficient if that target were repeatedly presented at the same location during training (single-target training, STT) as illustrated in Figure 3. This inefficiency arises because the motion experienced during training does not coincide with the motion that is to be learned, resulting in limited overlap between the motion-referenced learning that occurs and the learning that is desired.

The aforementioned inefficiency can be ameliorated by a paradigm which continually changes the locations of the targets presented during the training period as shown in Figure 3, second column. In this training paradigm, target directions would be shifted from one trial to the next so that the actual motion experienced repeatedly lines up with the motion to be learned. For the CW FF depicted in Figure 3, this corresponds to left-shifted training (LST). Initial target locations are placed with large leftward shifts with respect to the desired learning direction – in anticipation of the large rightward initial errors with respect to the target location. These leftward target shifts are then gradually reduced as learning proceeds and errors become smaller, in order to maintain alignment between the actual motion experienced and the movement to be learned.

The MRL hypothesis predicts that the LST training paradigm should produce faster learning than the standard STT paradigm used in previous motor adaptation studies in which a single target direction was trained [24], [26]. We tested this idea by comparing the learning curves associated with these training paradigms for adaptation to a clockwise viscous curl force-field. A different group of subjects was studied on each paradigm to avoid the effects of savings [27]–[29]. As a control for a possible increase in attention associated with changing target locations in the LST paradigm, we tested a third group of subjects with a right-shifted training (RST) paradigm. Here targets were shifted to the right, mirroring the target positions in the LST paradigm. The MRL hypothesis would predict slower learning for RST than STT or LST because right-shifted targets in a rightward pushing force-field would result in reaching movements even farther away from the desired learning direction than those expected in STT (see Figure 3, third column). In contrast the PRL hypothesis would predict fastest learning for the STT paradigm and identical learning rates for the LST and RST paradigms because the STT paradigm creates perfect alignment between the desired learning and the planned motion whereas the LST and RST paradigms create misalignments between the desired learning direction and planned motion that are opposite in direction but equal in magnitude. We used a FF magnitude of 22.5 N/(m/s) for these experiments – 2.5 times the magnitude used in Experiment 1 – in order to magnify the various misalignments discussed above. In all three paradigms, we measured learning at the desired learning direction (90°) by pseudo-randomly interspersing 90° error-clamp trials among the training trials with an average frequency of 20%.

**Figure 4:**
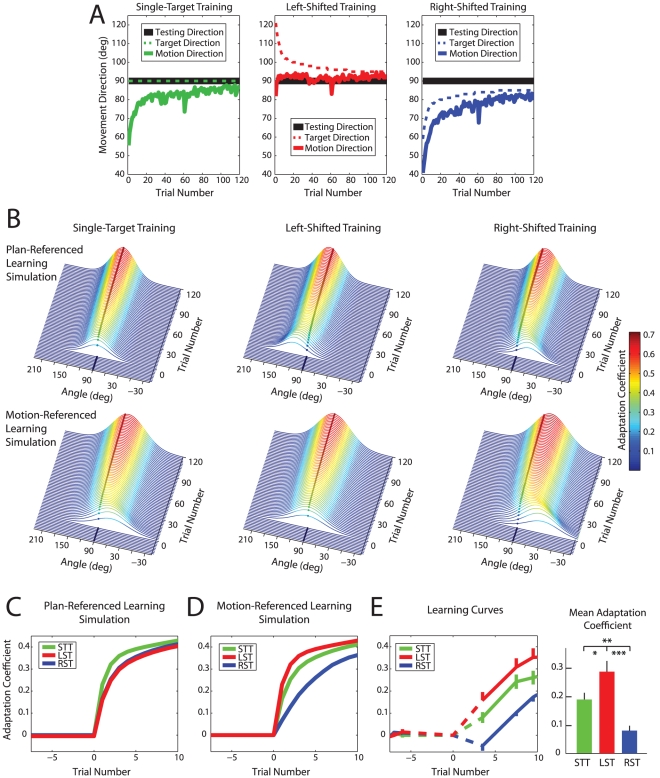
A novel training paradigm improves learning rates. (**a**) Characterization of the STT, LST and RST training paradigms. Target directions (dashed) and actual movement directions (solid) during the training period are plotted against trial number. Note that the LST paradigm achieves actual movement directions that are much more closely aligned with 90° than the other two paradigms. (**b**) Simulations of motor adaptation based on the PRL and MRL hypotheses for the three training paradigms. The darkened dots at 90° indicate the desired learning direction and the coloring indicates the amount of adaptation predicted. Note that PRL predicts optimal alignment with STT while MRL predicts optimal alignment with LST. (**c**) and (**d**) Predicted learning at 90° for the PRL and MRL hypotheses. Note that these traces represent slices at 90° through the 3-D plots in panel (b), corresponding to the darkened dots. (**e**) Experimental results for all three training paradigms. Note that over the first 10 trials, the LST paradigm produces the highest adaptation levels, and RST the lowest, as predicted by MRL. The error bars represent standard errors.

We first collected data from a subset of subjects in the STT paradigm in order to estimate the evolution of directional errors across trials. We used this pattern of directional errors to determine the target shifts that would produce good alignment between experienced motion and desired learning direction for the LST paradigm (see Materials and Methods). As shown in Figure 4A, we obtained a good match between motion direction and the desired learning direction (90°) throughout the training period for the LST paradigm, so that misalignment between these directions was dramatically reduced compared to the STT paradigm. Correspondingly, the misalignment between motion direction and the desired learning direction was about twice as great for RST than for STT.

The plots shown in Figure 4B illustrate how the adaptation patterns predicted by MRL and PRL would evolve as training proceeds for the training paradigms discussed above. Note that adaptation spreads across a limited range of movement directions consistent with local generalization [24]–[26], but the alignment between adaptation and the desired learning direction (90°) varies from one paradigm to another (STT vs. LST vs. RST), and from one credit assignment hypothesis to another (PRL vs. MRL). The darkened dots which highlight a slice through these plots at 90° illustrate the amount of adaptation associated with the desired learning direction.

These simulations show that the PRL hypothesis predicts that in the STT paradigm, credit assignment will be perfectly aligned with the desired learning direction (90°) throughout training. PRL also predicts an equal but opposite pattern of misalignments between credit assignment and desired learning for the LST and RST paradigms (Figure 4B). These misalignments are initially large but become attenuated during the course of the training because planned and actual motions converge. This results in simulated learning rates that are highest for the STT paradigm and lower, but identical, for the LST and RST paradigms under PRL (Figure 4B–C). In contrast, the simulations for the MRL hypothesis show perfect alignment between the credit assignment and the desired learning direction for the LST paradigm. For STT, the MRL-based simulations show a gross misalignment between the credit assignment and the training direction. For RST, the misalignment is even greater (Figure 4B). This results in learning rates that are predicted to be greatest for the LST paradigm, followed by the STT and RST paradigms, respectively (Figure 4B, D). As with the PRL simulations, the misalignments become attenuated as training proceeds.

### Left-shifted training improves learning rates

Our experimental data show a clear difference between the learning curves obtained for the three training paradigms in the early stages of training (first three EC trials; one-way ANOVA, *F*(2,87)  = 14.57 , *p*<4×10^−6^). The LST group displays the highest adaptation levels and the RST group displays the lowest adaptation levels as shown in Figure 4E. In particular, the LST group displayed an 86% increase in adaptation levels on the first EC trial and a 52% increase over the first three EC trials, whereas the RST group displayed a 59% decrease in adaptation levels compared to STT over the first three EC trials in the training period. Post-hoc comparisons between groups over the first three EC trials indicate that the LST group showed significantly greater learning than the RST group (*t*_58_ = −5.05, *p*<3×10^−6^). This result is in keeping with the MRL prediction, but defies the PRL prediction of equal learning rates for these groups. Our data also shows that the LST group displays significantly greater learning than the STT group (*t*_58_ = −2.17, *p*<0.02), in keeping with the MRL prediction, but opposing the PRL prediction of a greater learning rate for STT. We also find that the STT group displays significantly greater learning than the RST group (*t*_58_ = −3.90, *p*<2×10^−4^), corroborating the group order predicted by the MRL hypothesis. These findings provide additional support for motion-referenced learning and demonstrate that a training paradigm that is designed to leverage knowledge about the mechanism for credit assignment can improve learning rates compared to standard training procedures.

Inspection of the learning curve for the RST group reveals that the adaptation for the first EC trial after exposure to the FF actually dips a bit below zero. MRL predicts reduced learning for this group but would not predict opposite learning, consistent with the finding that the adaption level at this point, although nominally less than zero, is not significantly so (*t*_27_ = −2.01, *p*>0.05). Additionally, we note that the third-to-last error-clamp trial in the baseline (which is illustrated along with the full learning curve in Figure S2) displays an adaptation coefficient which dips below the average baseline and falls within the error bars of the first point in the RST learning curve, suggesting that the latter is not entirely outside the range of the data. Despite the differences in learning rate predicted by MRL-based credit assignment, angular errors should decrease as the training period proceeds. This results in reduced misalignment between prescribed and actual motion directions for the STT and RST groups, leading to a predicted convergence of the adaptation levels for all three groups (see Figure S2 and Text S1). Our data bears out this prediction: despite significant differences between groups early in the training period, we find no significant difference between groups late in the training period (last three EC trials; one-way ANOVA, *F*(2,87)  = 0.23, *p*>0.05). In addition, although we have shown that the MRL-based training paradigm (LST) increases the rate of adaptation, our results do not provide any information on the long-term retention for this adaptation. Further studies would be required to assess if the retention of the motor memories acquired using an MRL-based training paradigm is greater than that of memories acquired using single-target training paradigms.

## Discussion

Elucidating how associations are modified during the process of learning is a key step towards understanding the mechanisms underlying behavioral plasticity. Our findings demonstrate that the effect of the adaptation arising from an error sensed on a previous movement is greatest when the plan for the current motion matches the motion experienced on the previous trial. This indicates that, in the motor adaptation task we studied, the learned association binds the adaptive change in motor output to the actual motion experienced. We first showed that this motion-referenced learning hypothesis is able to explain the complex pattern of generalization that emerges when subjects are exposed to multiple blocks of interfering force-fields. We then followed up this result by showing that a manipulation of the pattern of target locations that aligned the actual motion experienced during training resulted in significantly improved learning rates, whereas a manipulation which increased misalignment resulted in significantly reduced learning rates. Together, these findings provide compelling evidence that credit assignment during motor adaptation is referenced to actual motions experienced rather than planned motions, and that this knowledge can be leveraged to improve the efficiency of motor skill training. The most general view of credit assignment would be that error-dependent motor adaptation might be composed of both motion-referenced and plan-referenced components. Although previous work overwhelmingly assumed pure plan-referenced learning [4], [9]–[16], our results indicate that motor adaptation is primarily composed of motion-referenced learning - in fact, our results are consistent with motor adaptation being fully motion-referenced. However, we cannot rule out a small contribution from plan-referenced learning. Consequently, further work will be needed to more precisely determine the relative contributions of each mechanism and to determine whether situations exist in which the levels of plan-referenced learning are substantial.

### Previous assumptions about credit assignment: plan-referenced learning

Despite the lack of direct evidence in support of it, plan-referenced learning has been widely assumed in the motor adaptation literature, particularly in modeling work in which a credit assignment scheme must be chosen, even if implicitly so, in order for a learning rule to be defined [4], [9]–[16]. Interestingly, Wolpert and Kawato (1998) assumed a hybrid credit assignment scheme: PRL for inverse-model learning and MRL for forward-model learning [4]. In principle, PRL is attractive because adaptation referenced to the previously planned motion would have the greatest effect on the same movement if it were repeated. In fact, Donchin et al. (2003), which models motor adaptation with a PRL learning rule, contains what the authors maintain is a proof that PRL-based learning is optimal in their supplementary materials [10]. However inspection of this proof reveals that its derivation is based on the assumption that motor adaptation acts to maximize the benefit that would be accrued if the same movement were repeated. In other words, this proof investigated what the optimal credit assignment procedure should be for STT and found that PRL maximizes the benefit of motor adaptation for STT. Since PRL is optimal for STT, MRL must be suboptimal for STT (as our simulations predict; see Figure 4B–D). This suggests that some training procedure other than STT would be optimal for MRL, and our data show that, for a clockwise FF, left-shifted training (LST) is indeed more effective than STT. Effectively, Donchin et al. (2003) assumed that a credit assignment procedure optimized for performance on STT would be used by the nervous system. Here we show that this is not the case. Instead, the error-dependent learning that occurs on a particular trial is referenced to the actual motion experienced on that trial rather than the planned motion, and as a result, STT produces slower learning than another training procedure (LST). Thus the human motor system does not adapt with the mechanism that would have the greatest effect on the same movement if it were repeated. Why would this be?

### Plan-referenced learning can lead to instability

The problem with PRL is that the dynamics experienced are generally functions of actual rather than planned motion. For example, the dynamics experienced from moving a small dense mass would be proportional to the actual rather than the planned acceleration of that mass. Note that the dynamics that subjects experienced in our experiments were also dependent on the actual motion state, i.e., the force was based on the velocity of the actual rather than the planned motion. The key consequence of this state dependence is that since the force pattern experienced during a particular motion does not reflect the planned motion (because it reflects the actual motion), the force pattern that would have been experienced if the planned motion were achieved is unknown. This means that, in principle, the error between the current motor output and the environmental dynamics acting on the planned motion adaptation is also unknown. Because this error is unknown, no learning rule for adaptation referenced to the planned motion can be guaranteed to reduce it. If, however, errors are small enough so that the dynamics experienced in actual and desired trajectories would be very similar to each other, plan-referenced learning schemes could converge because these schemes essentially assume equality between these dynamics. On the other hand, if errors are sufficiently large, using such a credit assignment scheme might result in unstable learning which does not converge on the desired motor output. Clearly, a credit assignment scheme that could lead to instability would be a liability for the CNS.

### The consequences of motion-referenced learning

The state dependence of physical dynamics insures that the force pattern experienced corresponds to the actual motion. Thus the error between the motor system's current estimate of the dynamics associated with the actual motion and the environmental dynamics associated with this motion can be determined. Because the motor output error corresponding to the actual motion can be determined, the motor output associated with it can be modified to reduce this error reliably, allowing for stable convergence of the motor output on the true environmental dynamics. This corresponds to motion-referenced learning. Interestingly, this reasoning is reflected in learning rules with mathematically provable stability that are widely used for the estimation of environmental dynamics in robotics and machine learning [17]–[21], [30]. These learning rules must be motion-referenced in order for stability to be assured.

One unfortunate consequence of motion-referenced learning is the suboptimal rate of motor adaptation observed if an individual were to repeatedly invoke the same motor plan when attempting to learn a novel task [30]. We demonstrate this suboptimality in the single-target training (STT) paradigm in Experiment 2 (Figure 4). Since adaptation proceeds according to the actual motion (rather than the planned motion), the STT paradigm leads to adaptation that is not aligned with the desired learning direction so that adaptation proceeds at a slower rate than if the actual motion is aligned across trials as in the LST paradigm. Our finding of motion-referenced credit assignment during motor adaptation is, therefore, compatible with the idea that the CNS favors a stable learning algorithm (MRL) over one that maximizes the effect of learning if the same motion plan is repeated at the expense of stability (PRL).

### The relationship between the magnitude of error and the amount of adaptation

Recent studies have provided evidence for reduced learning rates for large errors [31]–[33]. One of these studies proposed the rationale that this occurs because the motor system sees large errors as less relevant than small errors [31]. However, note that in these studies the adaptation was measured not along the motion direction experienced during the training trials, but along the direction of the previously planned movement – equivalent to STT. Therefore the decreased learning rates associated with large errors observed in these studies may be, at least in part, due to misalignment in motion-referenced credit assignment, because larger errors lead to increased misalignment between desired and actual motion during adaptation. This results in a corresponding misalignment between credit assignment and the desired learning, as illustrated in Figures 3 and 4. Further work will be required to determine the extent to which the apparent reduction in learning rates that has been observed with large errors reflects this misalignment versus a true reduction in the ratio between the amount of adaptation and the size of the error.

### The relationship between use-dependent learning and motion-referenced learning

A recent study by Diedrichsen et al. [34], provides evidence for the occurrence of use-dependent learning alongside error-based learning in reaching arm movements. This use-dependent learning describes a mechanism by which the trajectory of motion in task-irrelevant dimensions is gradually adapted to resemble the motion experienced on preceding trials. Therefore, use-dependent learning resembles motion-referenced learning in the respect that they both depend on the actual motion experienced. However, as noted by Diedrichsen et al. [34], use-dependent learning is oppositely directed from motion-referenced error-dependent learning when a perturbing force is experienced. This is because use-dependent learning would act to increase the extent to which future motions resemble the perturbed movement whereas (motion-referenced) error-dependent learning acts to oppose the effect of this force in order to reduce the extent to which future motions resemble the perturbed movement. A second key difference is that use-dependent learning is readily observed along task-irrelevant dimensions, but is either greatly reduced or entirely absent along task-relevant dimensions [34], whereas the motion-referenced learning that we demonstrate in the current study acts primarily along task-relevant dimensions in which error can be readily defined.

Taken together, the identification of motion-referenced learning and use-dependent learning expand what we know about the role of sensory information in motor adaptation, in particular sensory information about motion. In addition to the role that this sensory input plays in computing motor errors, the motion-referenced learning and use-dependent learning mechanisms respectively explain how sensed motion is specifically associated with error-dependent changes in motor output to reduce the difference between plan and action, and how sensed motion can be used to adapt which motions are planned to begin with.

### Sensed versus predicted motion and Bayesian estimation

Information about actual motion states is required for motion-referenced learning. This information can be acquired from delayed sensory feedback or estimated in real time through the use of a forward model, relying on an efference copy of the motor command and past sensory information [4], [35]–[39]. However, since sensory feedback signals and efference copy are noisy, actual motion must be estimated from imperfect information. Several studies have shown that the motor system integrates prior expectations about motion with noisy sensory feedback in order to estimate actual motion in accordance with Bayes Law [40]–[42]. The influence of prior expectations should increase with the level of sensory feedback noise, and so Bayesian estimation should have greater effects on motion estimation and thus on motion-referenced adaptation when noise levels are high.

### Motion-referenced learning in the adaptation to visuomotor transformations

What is the role of motion-referenced learning in the adaptation to a visuomotor transformation, where there is a dissociation between the actual motion of the hand and the actual motion of the cursor? A definitive answer to this question will require further experimental work since the present study looks at the adaptation to new physical dynamics rather than visuomotor transformations. A priori, it would seem that for visuomotor transformations, learning should be associated with the actual motion of the controlled object (cursor) rather than with the actual motion of the body part that is exerting this control. If motor learning were associated with the actual body motion, it would be difficult to see how large visuomotor rotations could be learned at all, because even late in adaptation, an arbitrarily large mismatch would exist between the planned motion (e.g., the motion of the cursor to its target position) and the actual hand motion. However, previous studies have shown that visuomotor rotations that are wider than the half-width of the generalization function for visuomotor rotation learning (about 30°) are readily learned [43]–[44]. A second point is that since (a) the motor planning during visuomotor transformation learning corresponds to the planned motion of the cursor (rather than the hand), and (b) the relevant motor errors involve the relationship between actual and planned or actual and predicted cursor movements (rather than hand movements) [43], [45], it would seem logical that the learning resulting from errors in this task would be associated with the cursor as well.

### Implications of motion-referenced learning for savings

Linear state-space models with multiple time courses of adaptation [28], [46] have been invoked as an explanation of savings – the phenomenon that describes the increase in learning rate when an adaption is relearned compared to the initial learning. However, even when complete behavioral washout of the learning is achieved, there appears to be some capacity for savings [29]. This effect cannot be captured by the aforementioned linear models, leading to the suggestion that significant nonlinearities arise even in simple motor adaptation experiments [29]. However, motion-referenced learning provides another possible explanation: Savings after washout may be due to a mismatch between the actual movement directions experienced in the initial learning and the washout trials rather than nonlinearities in the learning process. Such a mismatch would result in incomplete washout in the actual movement directions experienced during initial learning – similar to the residual direction-dependent adaptation that we demonstrate in Experiment 1. Further work will be necessary to determine the extent to which this is the case, but if savings after washout resulted in part from a directional mismatch during washout, then the prediction would be that the amount of savings would be reduced if the washout trials spanned the movement directions experienced early in training, rather than being confined to a single target direction as in [29].

### The relationship between cerebellar physiology and motion-referenced learning

Studies with healthy subjects [47]–[48] and subjects with congenital and acquired cerebellar deficits [49]–[51] have provided evidence that the cerebellum participates in motor adaptation. It has been proposed that the simple spike firing of Purkinje cells in cerebellar cortex contributes to motor output and that error signals carried by climbing fibers modify the strength of the parallel fiber synapses onto Purkinje cells [11], [48], [52]–[53]. This plasticity alters the effect that the information carried in parallel fibers has on the output of Purkinje cells, and thus on motor output [52]–[53]. Since parallel fibers carry sensory feedback (amongst other) signals [38], [54]–[55], this plasticity alters the association between sensory feedback about the actual motion and future motor output and may represent a neural mechanism for motion-referenced learning.

### Using knowledge of credit assignment during motor adaptation to improve neurologic rehabilitation

A common technique in neurorehabilitation is the use of partial assistance, where a therapist or device supplements movement in order to allow patients to better approximate a desired motion [56]–[58]. Since partial assistance reduces the difference between the actual and desired motions, our findings would suggest that it improves the alignment between the adaptation that is learned and the desired motion that is being trained. This would improve the efficiency of the training procedure. However, partial assistance would also reduce the magnitude of the motor errors that drive learning. These opposing effects may decrease the overall benefit of this procedure.

Interestingly, a method known as error augmentation that can be thought of as essentially the opposite of partial assistance has recently been proposed as a means to improve the rate of motor learning during rehabilitation. In error augmentation, motor errors are increased beyond normal levels by transiently exposing patients to perturbations that are stronger than those that are to be learned [59]–[61]. The rationale behind this technique is that since error signals drive motor learning, increasing the size of this signal may improve the rate of learning. Our results indicate that, like partial assistance, error augmentation will result in two opposing effects. Whereas, partial assistance increases the alignment between the motion-referenced learning which will occur and the desired learning but reduces the magnitude of the error signal driving adaptation, error augmentation decreases the alignment between the motion-referenced learning which will occur and the desired learning but increases the magnitude of the error signal driving adaptation. Thus, unlike partial assistance, error augmentation may provide a robust error signal for learning, but could in fact lead to decreased learning rates by magnifying the misalignment between the desired motion to be learned and the learned motion in the experienced trials.

The problem of opposing effects resulting from both of these training procedures could potentially be solved by the implementation of a training procedure analogous to the LST training we studied which aligned actual and desired movements, but with stronger-than-normal perturbations. Note that the design and implementation of a training procedure that aligns actual and desired motions is somewhat challenging. Even for the simple planar point-to-point movements we studied in Experiment 2, we first ran another group of subjects to determine the magnitude of the target shifts employed in each trial of our LST paradigm. For training more complex natural motions the challenge will be even greater. With higher-dimensional complex movements, simple manipulations like the altered target position we used in our LST paradigm might not be nearly as effective as a more complex manipulation like the imitation of the entire time course of an altered motion in providing good alignment between actual and desired movement. However, if a training procedure can be created that improves the alignment of the actual motions experienced with the desired motion, even when motor errors are large, such a paradigm may be capable of simultaneously benefitting from increased error-dependent learning and improved transfer of adaptation to the desired motion – the best of both worlds from error augmentation and partial assistance.

The improvement afforded by the LST paradigm or derivatives of it might even be more substantial if used in patients undergoing neurorehabilitation. For example, chronic stroke patients are able to adapt to dynamic environments, but display slower learning rates and higher residual errors than healthy controls [62]–[63]. Interestingly, our modeling efforts suggest that MRL-based training would have an even greater effect on subjects with these types of impairments, with the advantage of LST over STT predicted to be greater in magnitude and longer lasting as shown in Figure S2, because the higher motor errors these subjects normally experience lead to greater-than-normal misalignment under STT (see Text S1). Further studies would be required to determine whether an MRL-based training paradigm could lead to clinically significant improvements in neurologically impaired subjects.

## Materials and Methods

### Ethics statement

All experimental participants were naïve to the experimental purpose, provided informed consent and were compensated for their participation. All the experimental protocols were reviewed and approved by the Harvard University Committee on the Use of Human Subjects in Research (CUHS).

### General task description

Subjects performed 10 cm reaching movements in the horizontal plane with their dominant hands while grasping the handle of a 2-link robotic manipulandum. Subjects were seated with their forearm leveled with the robotic manipulandum and supported by a sling. The subjects were presented with 1 cm-diameter circular targets displayed on a vertically oriented LCD monitor. The position of the subject's hand was represented on the LCD monitor by a 3 mm cursor. Position, velocity and force at the handle were measured with sensors installed in the manipulandum at a sampling rate of 200 Hz. The subjects were instructed to produce fast, continuous movements, and were provided visual feedback throughout the movement. Feedback about the movement time achieved was presented at the end of each movement. Ideal completion times (500±50 ms) were signaled by an animation of the target while a chirp sound was played. For movement completion times that were below or above the ideal range the targets were colored blue and red, respectively. The mean peak speed for the movements in all experiments was 0.302±0.017 m/s. In certain movements, the subjects' trajectories were perturbed by velocity-dependent dynamics. This was implemented by a viscous curl force-field at the handle produced by the motors of the manipulandum, Equation 1.
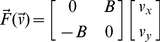
(1)


In this equation the constant *B* represents the viscosity associated with this force-field and has units of N/(m/s). Note that the direction of the force is always orthogonal to the direction of the velocity vector. We assessed the level of adaptation using methods described elsewhere [28]. Briefly, we measured the force pattern that subjects produced when their lateral errors were held to near zero values in an error-clamp [28], [64]–[65]. We then regressed the measured force pattern onto the ideal force required to fully compensate for the force-field. The slope of this regression was used as the adaptation coefficient that characterized the level of learning. For a force profile that is driven by adaptation to a velocity-dependent force-field, our adaptation coefficient represents the size of the bell-shaped velocity-dependent component of the measured force profile. This velocity-dependent component of the measured force profile specifically corresponds to the force component targeted to counteract the velocity-dependent force-field perturbation.

### Experiment 1: Generalization after force-field interference training

Twenty-eight individuals with no known neurologic impairment (mean age  = 19.9±1.8 years; 15 male) were recruited for this experiment. The first twelve subjects practiced the reaching task in 9 different directions (*θ* = 180°, 210°, 240°, 245°, 270°, 285°, 300°, 330°, 360°) for 254 movements (baseline), and were then trained to compensate velocity-dependent force-fields in a particular movement direction (270°) for 672 movements (training) with the direction of the FFs alternating every 7±2 movements between CW (B = 9 N/(m/s)) and CCW (B = −9 N/(m/s)). Thus the ratio of CW to CCW FF trials was 7∶7. After blocks of 168 training (FF) trials, the pattern of generalization was measured in each direction during a testing block of 40 consecutive EC trials spread across these directions. The direction (CW or CCW) of the last FF presented before generalization testing was balanced across the four training blocks.

A second group of subjects performed the same experiment but with different baseline/testing directions (*θ* = −30°, 0°, 30°, 45°, 60°, 75°, 90°, 120°, 150°) and training direction (60°). In this experiment the ratio of CW to CCW FF trials was 6∶8 for the first six subjects and 5∶9 for the subsequent ten subjects. The data from the subjects trained at 270° and that from the last ten subjects trained at 60° (CW to CCW FF trial ratio of 5∶9) are shown in Figure 2C. The CW to CCW FF ratio was adjusted to eliminate the bias towards learning the CW FF we observed in the first 12 subjects – details are provided in Text S1. The data for the subjects with the 6∶8 CW to CCW FF trial ratio are compared to the other datasets in Figure S1.

### Experiment 2: Comparison of training paradigms

Ninety individuals with no known neurologic impairment (mean age  = 22.0±5.9 years; 44 male) were recruited for this experiment. One group of subjects (N = 30) were assigned to the single-target training (STT) paradigm. Here the subjects performed 75 movements in a single direction (90°) to practice the reaching task (baseline) and then were exposed to a CW velocity-dependent force-field (CW; B = 22.5 N/(m/s)) for 125 reaching movements to the same direction (training). The learning level during baseline and training was assessed with randomly interspersed EC movements (*p*(EC)  = 0.2). The mean trial history of angular errors 300 ms into the movement during force-field trials was obtained for this group of subjects and used to design the left-shifted (LST) and right-shifted (RST) training paradigms.

In the LST paradigm, the directions of the reaching targets were adjusted by adding a smoothed fit of the mean trial history of angular errors from the first seventeen subjects of the STT experiment to the desired learning direction on the corresponding trial (90°). We did this so that when subjects reached to these shifted targets their actual motion would be expected to line up with the desired learning direction if the directional error on that trial was similar to that observed in the STT group as illustrated in Figure 3. On the other hand, in the RST training paradigm we subtracted this trial history of angular errors from the STT experiment to the desired learning direction (90°). Therefore these target locations mirrored the LST target locations across 90°. We did this so that when subjects reached to these shifted targets their actual direction of motion would be deviated twice as much from the desired learning direction (90°) as in the STT experiment. In the LST and RST paradigms subjects (30 on each group) also performed 75 baseline movements and then performed 125 training movements using the same CW velocity-dependent FF that was learned by the STT paradigm group. The learning level during baseline and training was assessed by measuring the lateral force profiles produced during randomly interspersed EC trials (*p*(EC)  = 0.2) directed toward the desired learning direction (90°).

We simulated the adaptation process for the STT, LST, and RST training paradigms for the PRL and MRL credit assignment schemes using the model equations and parameters described below and in Text S1. However, in this case, since the experiments and simulations were not aimed at assessing generalization, error in the simulations was defined as the difference between the desired adaptation in the target direction and the actual adaptation in that direction.

### Modeling and simulation of credit assignment mechanisms

We simulated the adaptation process predicted by PRL and MRL for both experiments. We used linear state-space models [28] with local motor primitives to model the adaptation and its generalization (see Text S1 for details). These are discrete (trial-dependent) error driven models, where the error is calculated as the angular difference between the planned movement direction and the actual movement direction, Equation 2.
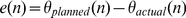
(2)


In the learning rules presented in Equations 3 and 4, the adaptation, *x*, for given movement direction, *θ* (*θ* can take on values encompassing the entire movement space), in a given trial, *n + 1*, is the sum of the previous adaptation level for the same movement direction weighted by a retention coefficient, *A*, and the learning occurring in the current trial which is given by the product of the error in the current trial and a local motor primitive, *B*. For the PRL model (Equation 3), this local motor primitive, *B*, is centered at the planned movement direction, *p_lanned_*, implying that after a given trial, the maximum adaptation in the entire movement space occurs at the planned movement direction.
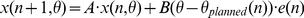
(3)


Alternatively for the MRL model (Equation 4), the local motor primitive is centered at the actual movement direction, *a_ctual_*, which implies that after a given trial, the maximum adaptation occurs along the actual movement direction.
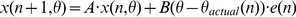
(4)


### Data inclusion criteria

In our data analysis a few grossly irregular trials were excluded. This included movements that were extremely fast (peak velocity >0.55 m/s) or extremely slow (peak velocity <0.2 m/s), as well as trials with extremely fast (<75 ms) or extremely slow (>2.5 sec) reaction times. This insured that subjects did not initiate movements too quickly, without correctly identifying the location of the target, or too late, indicating that they might have not been attending to the task. For Experiment 1, application of these two criteria resulted in the inclusion of 98.2% of the trials in the 270° group, 96.8% of the trials in the first 60° group (6∶8 CW to CCW FF trial ratio), and 94.9% of the trials in the second 60°s group (5∶9 CW to CCW FF trial ratio). For Experiment 2, 94.7% of the trials in the STT group, 95.2% of the trials in the STT group, and 93.4% of the trials in the RST group were included.

### Statistical analyses

In order to compare the predicted and experimentally observed generalization patterns in Experiment 1, we computed the correlation coefficient between them as well as the *p* value and *F*-statistic associated with the slope of the corresponding linear regression. We assessed the significance of the difference in the adaptation between the peaks of the generalization patterns using one-sided paired *t*-tests. In Experiment 2, differences between learning rates for the three training paradigms (STT, LST, and RST) were assessed with one-way ANOVAs both early (first 3 EC trials) and late (last 3 EC trials) in training. When significant differences arose, post-hoc comparisons were performed using one-sided *t*-tests.

## Supporting Information

Figure S1 Results of FF interference generalization experiments. (a) Generalization pattern for force-field interference experiment training at 270° (−90°). Notice that the adaptation is biased towards the CW FF (negative adaptation). This is apparent at the training direction (black circle). (b) Evolution of adaptation during replication experiment, training at 60° and using a CW to CCW FF trial ratio of 6∶8. Notice that the mean adaptation remains consistently biased towards the CW FF (black trace and black diamond for overall mean adaptation). (c) Evolution of adaptation during replication experiment, training at 60° and using a CW to CCW FF trial ratio of 5∶9. Notice that here the mean adaptation is not biased toward the CW or CCW FF (black trace and black diamond for overall mean adaptation). (d) Generalization pattern for the interference experiments with training at 60°. Notice that although the adaptation is biased towards the CW FF (negative adaptation) for a 6∶8 CW:CCW FF trial ratio (light grey trace), this bias is removed when the ratio is modified to 5∶9 (dark grey trace). All generalization patterns are consistent with the prediction of the MRL hypothesis (r>0.7 in all cases). The error bars represent standard errors.
(EPS)

Figure S2 Comparison of Different Training Paradigms. (a) Simulated learning curves for STT, LST and RST paradigms according to the MRL hypothesis during extended training – similar to 4D. (b) Experimental results showing the extended learning curves for the STT, LST and RST training paradigms. (c) Simulated learning curves for chronic stroke patients with reduced learning rates and higher residual errors (based on parameters from [62]–[63]) trained with the STT, LST and RST paradigms according to the MRL model. The error bars represent standard errors.
(EPS)

Text S1 Supporting Information. Contains an example of credit assignment for error-based learning, further details on the modeling and simulation of credit assignment for the different experiments, an explanation of the adjustment of force-field trial ratios in the force-field interference experiments, a discussion of the extended learning curves for the experiments comparing different training paradigms, simulations of learning for stroke patients according to these paradigms, and a review of previously published models with motion-referenced learning implementations.
(DOC)
